# Reformation of the Electron Internal Transport Barrier with the Appearance of a Magnetic Island

**DOI:** 10.1038/s41598-019-56492-x

**Published:** 2020-01-08

**Authors:** N. Kenmochi, T. Minami, T. Mizuuchi, C. Takahashi, G. M. Weir, K. Nishioka, S. Kobayashi, Y. Nakamura, H. Okada, S. Kado, S. Yamamoto, S. Ohshima, S. Konoshima, Y. Ohtani, K. Nagasaki

**Affiliations:** 1 Graduate School of Frontier Sciences, The University of Tokyo, Kashiwa, Chiba 277-8561 Japan; 2 Institute of Advanced Energy, Kyoto University, Uji, Kyoto 611-0011 Japan; 3 Max-Plank-Institute für Plasmaphisik, Tailinstitut Greifswald, EURATOM Association, Wendelsteinstr. 1, Greifswald, D-17491 Germany; 4 Department of Physics, Nagoya University, Nagoya, Aichi 464-8602 Japan; 5 Graduate School of Energy Science, Kyoto University, Uji, Kyoto 611-0011 Japan

**Keywords:** Magnetically confined plasmas, Plasma physics

## Abstract

When realising future fusion reactors, their stationary burning must be maintained and the heat flux to the divertor must be reduced. This essentially requires a stationary internal transport barrier (ITB) plasma with a fast control system. However, the time scale for determining the position of the foot point of an ITB is not clearly understood even though its understanding is indispensable for fast profile control. In this study, the foot point of the electron ITB (eITB) was observed to be reformed at the vicinity of a magnetic island when the island started to form. In addition, the enhanced confinement region was observed to expand during the eITB formation according to the radial movement of the magnetic island toward the outer region. Compared to the time scales of the local heat transport, the faster time scales of the movement of the eITB foot point immediately after island formation (~0.5 ms) suggest the importance of the magnetic island for plasma profile control to maintain stationary burning.

## Introduction

The key issues in realising future fusion reactors involve the maintenance of stationary burning and reduction of heat flux to the divertor with high-pressure core plasma^1–3^. For this, the stationary internal transport barrier (ITB) plasma, the pressure gradient of which sharply changes in the core region, and the fast control system of the ITB plasma profile are considered essential and effective. The time scale for determining the position of the foot point of an ITB is not clearly understood even though its understanding is indispensable for fast profile control. Therefore, a deeper understanding of the physical mechanism in determining the ITB profile, especially the radial propagation of ITB region, is necessary for maintaining stationary ITB plasma and profile control.

The formation of the electron ITB (eITB), especially the determination of its foot point, has been reported to be affected by the neoclassical transport and existence of a rational surface and magnetic island. In the Large Helical Device (LHD) experiments, the magnetic island located near the foot point of the eITB contributes to the formation of the eITB by producing radial electric field shear at the magnetic island boundary^4,5^. A similar mechanism showing the effect of a magnetic island on the plasma transport has also been observed through numerical simulations such as gyrokinetic calculations^6^. In ref. ^7^, the simulation showed that transport is reduced in the core plasma region adjacent to the magnetic island because of reduction in micro-turbulence, which is caused by the increase in flow shear around the magnetic island. In addition, the ITB location, at which a large temperature gradient appears, is found to be strongly correlated to the rational surface. Kishimoto *et al*.^8^ theoretically studied the ITG mode simulation to explain the reduction in transport near the minimum *q* area on the rational surface. This simulation predicts the suppression of turbulent transport around the minimum *q* surface due to the discontinuity of the phase relationship in the global wave structure across the minimum *q* surface. This discontinuity is more efficiently established with the increase in the flow shear and the decrease in the curvature of *q* at the minimum *q* surface^9^. In stellarator/heliotron plasmas, the eITB is thought to be characterised by the transitions of the neoclassical transport between the “ion root” (with a small magnitude of radial electric field (Er), usually negative) and the “electron root” (with a large positive Er); these are based on a bifurcation mechanism^10–13^. Although the effects of the neoclassical transport and existence of the rational surface and magnetic island for the eITB have already been investigated experimentally and theoretically, the interaction between these three mechanisms has not yet been investigated. The specific characteristics of Heliotron J, i.e., low magnetic shear and high flexibility, based on the magnetic configuration allow us to control low-order rational surfaces within the rotational transform profile, and therefore study how the magnetic topology affects eITB formation. Here we report the structure formation of the eITB region immediately after the formation of a magnetic island. We also discuss the expansion of the improved confinement region following the movement of the magnetic island.

## Results

### Observation of structural formation around the eITB foot point

**Figure 1:**
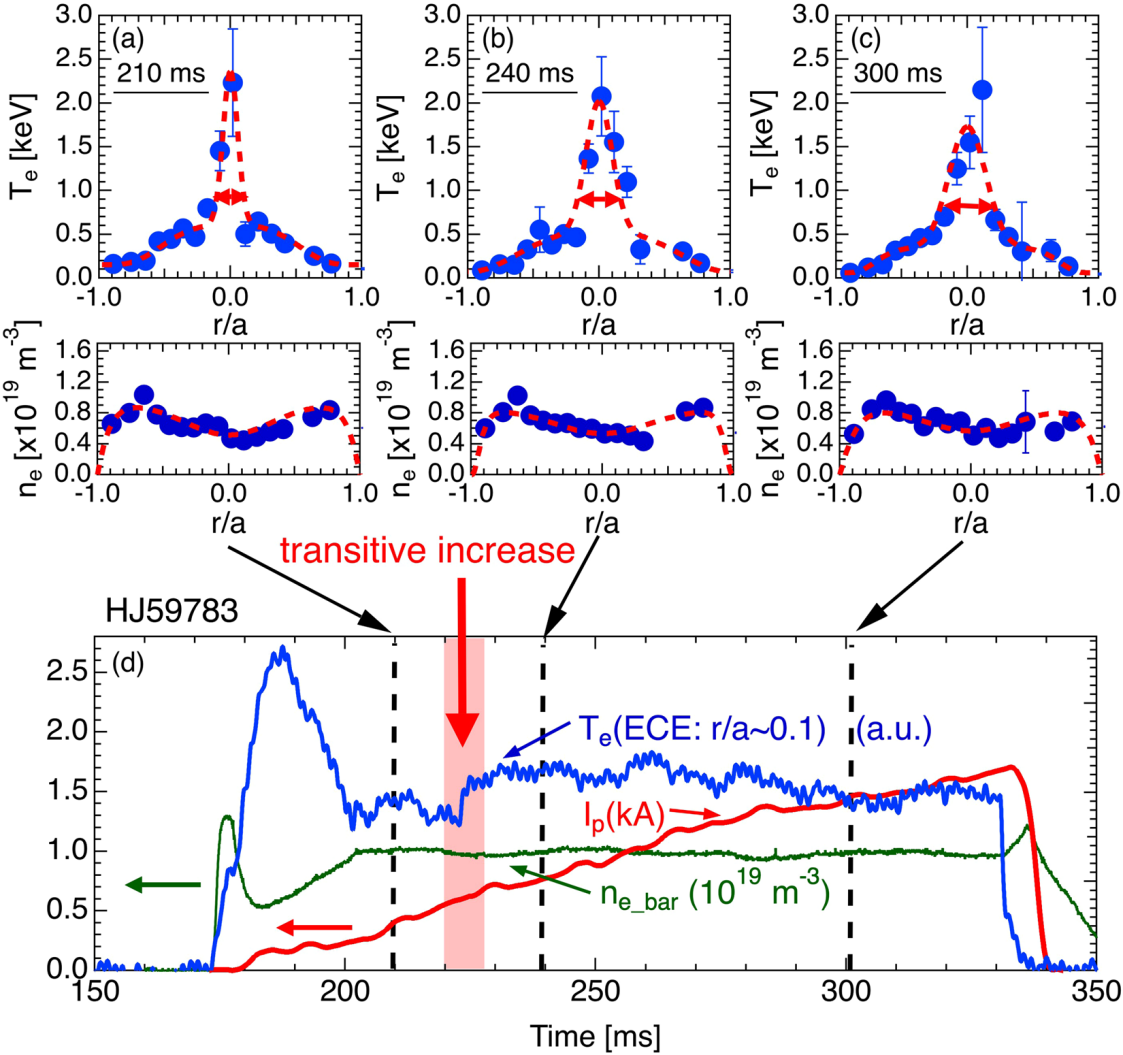
Rapid increase in *T*_*e*_ during eITB formation with current ramp-up. (**a**–**c**) *T*_*e*_ and *n*_*e*_ profiles. (**d**) Time evolution of *T*_*e*_, *n*_*e*_, and plasma current.

The effect of rational surface and/or magnetic island on the profiles of eITB plasmas was investigated in Heliotron J under constant electron density (*n*_*e*_), which fulfils the condition of low *n*_*e*_ (${\bar{n}}_{e} <  \sim \,1.2\times {10}^{19}{{\rm{m}}}^{-3}$) required to form the eITB^14^. Figure 1 shows the typical time evolution of electron temperature (*T*_*e*_), *n*_*e*_, and the plasma current as well as the *T*_*e*_ and *n*_*e*_ profiles measured through the Nd:YAG Thomson scattering (YAG-TS) system^15–17^ at 210, 240, and 300 ms. The eITB formation, which is characterized by a peaked *T*_*e*_ profile shape was observed from *t* = 200 to 330 ms. When the plasma current increased up to 0.7 kA at *t* = 223 ms, a sudden jump was observed in *T*_*e*_ at *r*/*a* ~ 0.1 in the electron cyclotron emission (ECE) signal. This increase does not represent the transition to the eITB formation because the eITB was already formed before the rapid rise in the ECE signal. The YAG-TS measurements show that the enhanced confinement region in the eITB expands at the transitive increase.

**Figure 2:**
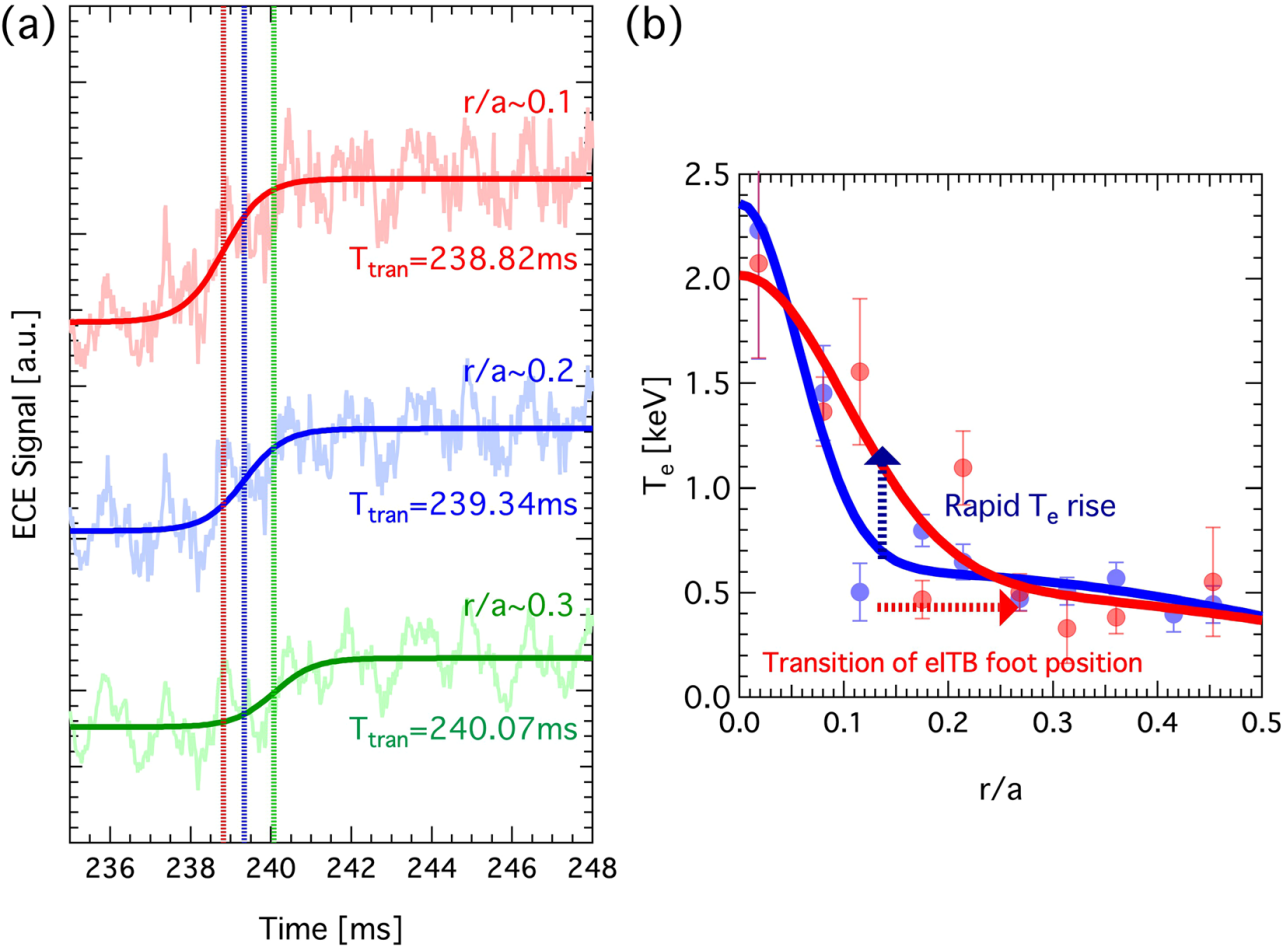
(**a**) ECE signals at *r*/*a* ~ 0.1, 0.2, and 0.3 during the rapid increase in *T*_*e*_. (**b**) *T*_*e*_ profiles before and after the rapid rise in *T*_*e*_.

The expansion speed of the enhanced confinement region was investigated using radially separated ECE channels. Figure 2(a) shows the ECE signals at *r*/*a* ~ 0.1, 0.2, and 0.3 during the rapid rise in *T*_*e*_ over ~2 ms. The transition times (*T*_*tran*_) of each point were estimated by fitting the curve of the ECE signals as follows:

1 $${S}_{ECE}(t)=A\,{\tanh }(t-{T}_{tran})+C.$$

The *T*_*e*_ profiles before and after the rapid rise in *T*_*e*_ are shown in Fig. 2(b). During the rapid rise in *T*_*e*_, the eITB foot point rapidly moved toward the outer region within several hundreds of microseconds, resulting in a delay in the rise of *T*_*e*_ toward the outer region. Here, the effective electron thermal diffusivity (${\chi }_{e}^{{\rm{eff}}}$) and the area of the magnetic–flux surface at *r*/*a* = 0.15 were approximately 3.5 m^2^/s and 1.2 m^2^, respectively. Therefore, the heat flux passed through the magnetic-flux surface at *r*/*a* = 0.15 in ~350 ms at a speed of ~0.1 m/s. The expansion speed of the eITB foot point was ~30 m/s, which is greater than that of the thermal transport. The power-deposition profiles of electron cyclotron resonance heating (ECH) calculated before and after the movement of the foot point using the TRAVIS code^18^ are both localised inside *r*/*a* = 0.1 with almost the same shape. Therefore, the transitive expansion of the enhanced confinement region by the eITB during the current ramp-up cannot be explained by the change of the ECH absorption because *T*_*e*_ and *n*_*e*_ at the resonance zone do not change. A “structural formation” around the eITB foot point is considered to cause the rapid movement of that foot point.

### Role of magnetic island in structural formation

**Figure 3:**
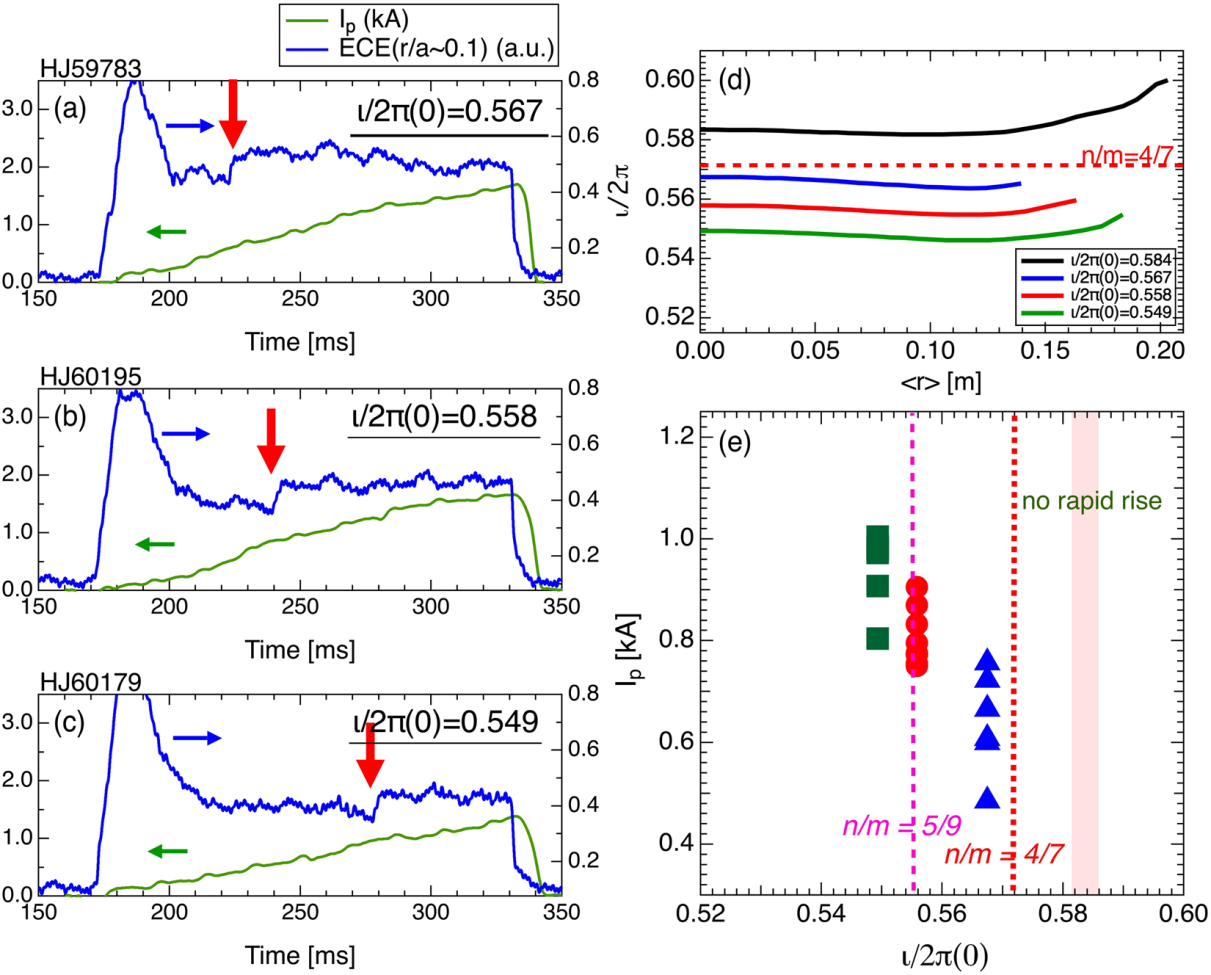
ECE signals and plasma currents for different rotational transform profiles: (**a**) $\iota /2\pi (0)=0.567$, (**b**) $\iota /2\pi (0)=0.558$, and (**c**) $\iota /2\pi (0)=0.549$. (**d**) Vacuum rotational transform profiles and (**e**) current at the start of the expansion of eITB as a function of $\iota /2\pi (0)$ for the vacuum magnetic field.

**Figure 4:**
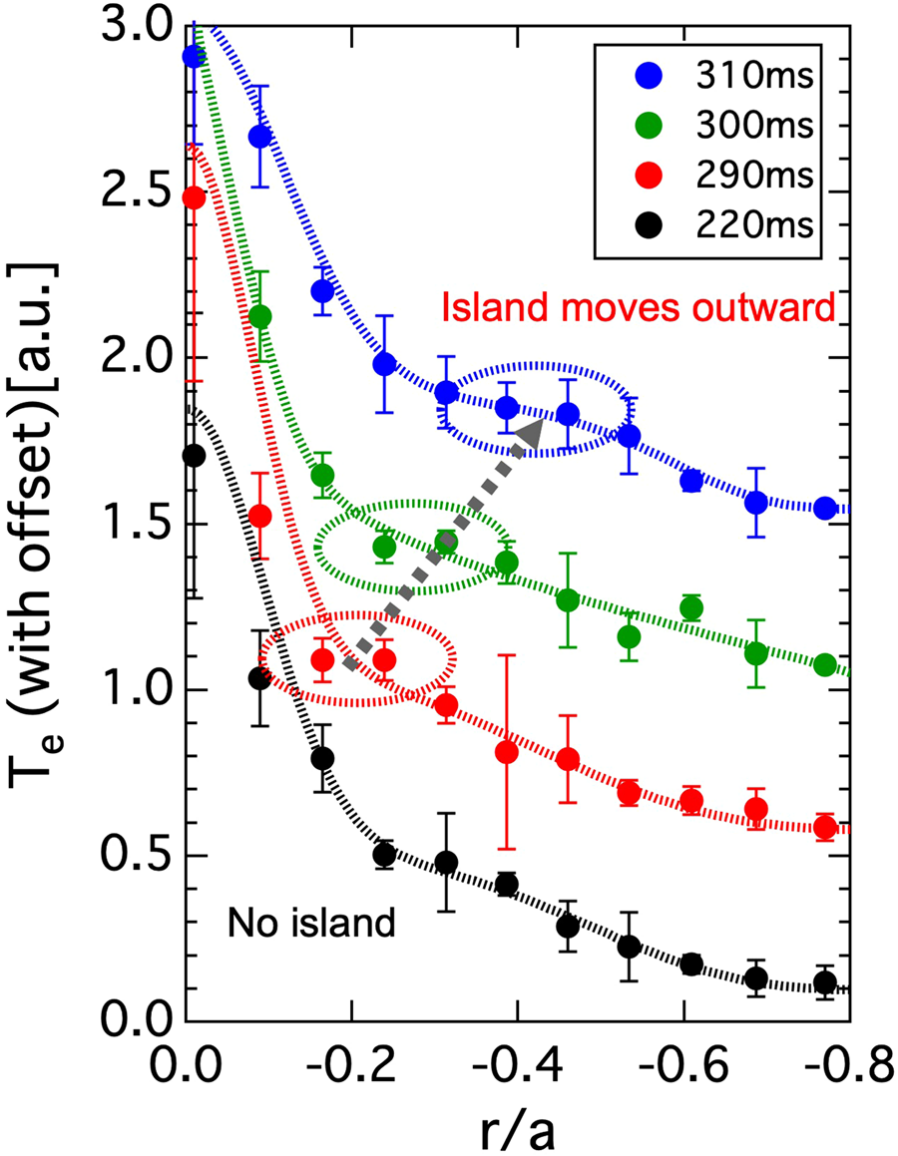
Radial movement of the island structure according to increase of toroidal current.

To clarify the role of the rational surface and/or magnetic island in the structural formation, the eITB formations for other magnetic configurations with rotational transform values of $\iota /2\pi (0)$ = 0.549, 0.558, 0.567, and 0.584 were investigated. The profiles of the rotational transform are shown in Fig. 3(d). In addition, the plasma with eITB was produced by a centrally focused 70 GHz ECH (*P*_*inj*_ ~ 270 kW). As the condition of the eITB formation depends on *n*_*e*_, *n*_*e*_ was controlled to be fixed through gas puffing for different magnetic configurations. Figure 3(a–c) show the ECE signals and plasma currents for the different rotational transform profiles. The structural formation was delayed with the decrease in the rotational transform values. In contrast, no structure was formed for the magnetic configuration of $\iota /2\pi (0)$ = 0.584. Figure 3(e) shows the current at the start of the expansion as a function of $\iota /2\pi (0)$ of the vacuum magnetic field. The required plasma current to the expansion decreased with the decrease in the rotational transform values except the rotational profile at $\iota /2\pi (0)$ = 0.584. The *n*/*m* = 4/7 rational surface is important because it is a candidate in which the magnetic island is produced owing to the *n* = 4 toroidal periodicity of the vacuum magnetic field of Heliotron J. The *n*/*m* = 4/7 is larger at $\iota /2\pi (0)$ = 0.567 and smaller at $\iota /2\pi (0)$ = 0.584. As the bootstrap current is driven in the direction of the rotational-transform increase, the 4/7th rational surface cannot be produced at $\iota /2\pi (0)$ = 0.584. Consequently, the small differences between the 4/7th rational surface and rotational transform values reduce the required plasma current for a structure formation, except at $\iota /2\pi (0)$ = 0.584. Although there exist other low-order rational surfaces (e.g., *n*/*m* = 5/9 ~ 0.556), at which the possibility of the formation of a magnetic island is low, around the *n*/*m* = 4/7 rational surface, the structural formation is only related to that particular rational surface. This result strongly suggests that the movement of the eITB foot point is affected by the existence of a magnetic island instead of a rational surface. Figure 4 shows the time evolution of *T*_*e*_ profiles at four time intervals (*t* = 220, 290, 300, and 310 ms) in the same discharge as shown in Fig. 3(c). The *T*_*e*_ profiles were observed to be flattened in the vicinity of the eITB foot point with increase in the toroidal current (see the profiles of *t* = 290, 300, and 310 ms). The radial position of the *T*_*e*_ flattening moves outward with the movement of the *n*/*m* = 4/7 rational surface. This movement is consistent with the calculated position of the *n*/*m* = 4/7 rational surface, suggesting the existence of a magnetic island. Note that we confirmed experimentally that the flat structure of *T*_*e*_ did not appear in plasmas without rational surfaces.

**Figure 5:**
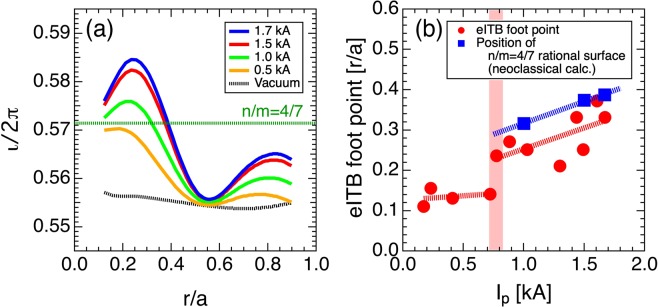
(**a**) Neoclassical calculation of rational transform profiles at *I*_*p*_ = 0.5, 1.0, 1.5, 1.7 kA, and vacuum. (**b**) Foot point of eITB and the position of *n*/*m* = 4/7 rational surface estimated through the neoclassical calculation as a function of plasma current. Transition occurs at ~0.7 kA.

The rotational transform profile, including bootstrap current for the magnetic configuration for which vacuum $\iota /2\pi (0)$ = 0.558, was calculated using the VMEC code, as shown in Fig. 5(a). The neoclassical bootstrap current profile at 210 and 300 ms was derived through the Sugama–Nishimura moment method, and is shown to be consistent with the experimental observation in Heliotron J^19,20^. The *n*/*m* = 4/7 rational surface was produced around *r*/*a* ~ 0.37 at ~1.5 kA (*t* = 300 ms). The calculation shows that the eITB can be formed without the rational surface. The formation time of the *n*/*m* = 4/7 rational surface is consistent with the structure formation time. The calculation also shows the outward movement of the eITB, which is synchronous with the movement of the rational surface with the increase in current. Figure 5(b) shows the eITB foot point, which is derived from the *T*_*e*_ profiles, and the positions of *n*/*m* = 4/7 rational surface estimated through neoclassical calculation as a function of the plasma current. Before the structure formation, the eITB foot point was kept at the same location. When the plasma current reached 0.7 kA, the location of the eITB foot point jumped to the outside of the plasma from *r*/*a* ~ 0.13 to *r*/*a* ~ 0.23. After the structure is formed, the location continued moving outward from *r*/*a* ~ 0.2 to *r*/*a* ~ 0.4 with the increase in current. The eITB foot points are located slightly inside the position of the *n*/*m* = 4/7 rational surface. However, the changes in the *n*_*e*_ profiles are insignificant during the discharge. Although the central *T*_*e*_ slightly decreases at the end of the discharge, the kinetic stored energy increases after the structure is formed owing to the expansion of the enhanced confinement area.

As described in the previous sections, the following three experimental facts provide an insight for one of the key issues in the fusion research which is whether a magnetic island or simply a low-*q* rational surface are the drivers of ITB formation/evolution^21–30^:

1. The *T*_*e*_ profiles, which represent the characteristics of the magnetic island, were observed to be flattened in the vicinity of the eITB foot point with increase in the toroidal current (See Fig. 4).
2. The eITB foot points are located slightly inside the numerically derived position of the *n*/*m* = 4/7 rational surface corresponding to the magnetic island formation around the rational surface and eITB formation at the inner boundary of the magnetic island (See Fig. 5).
3. The structural formation is only related to the particular rational surface at which the possibility of the formation of a magnetic island is high, although there exist other low-order rational surfaces (See Fig. 3).

These results suggest that the structural formation is affected by the magnetic islands, which is contradictory to consider as the rational surfaces.

## Discussion and Summary

The time scale of the structure formation compared to that of the local heat transport suggests that the structure formation is affected by the radial electric field, which can be formed transiently. Once a magnetic island is formed, a radial electric field is considered to form more rapidly than the heat transport in the vicinity of the magnetic island because of turbulence reduction, which is caused by the increase in the flow shear around the magnetic island. The resulting radial electric field affects the confinement improvement, resulting in the rapid movement of the eITB foot point.

In summary, we report on the structure formation at the eITB foot point in the vicinity of the magnetic island formed on Heliotron J. The experimental results show that (i) the foot point of eITB moves transitively outward when the island starts developing; (ii) after the structure formation, the foot point continues to move outward following the movement of the magnetic island and the improved confinement region expands. These results show a synergy effect of the eITB and magnetic island for the expansion of the improved confinement region. The time scales of the movement of the eITB foot point immediately after the island formation (~0.5 ms) are much faster than that of the local heat transport, suggesting the importance of the magnetic island for the plasma profile control to maintain stationary burning.

## Methods

### Heliotron J

The experiments were performed on Heliotron J, which is a medium-sized helical-axis heliotron device with a periodicity of (*l*, *m*) = (1, 4), where *l* and *m* are the pole number and pitch numbers of the helical coil, respectively. The major and averaged minor radii are 1.2 and 0.1–0.2 m, respectively^31^. The magnetic-field strength is 1.25 T and the working gas is deuterium. As the magnetic configuration of Heliotron J has low magnetic shear, the vacuum rotational transform profile is almost flat and the value of central $\iota /2\pi (0)$ is 0.558. The plasma with eITB was produced through centrally focused 70 GHz electron cyclotron resonance heating (ECH; *P*_inj_ ~ 270 kW, single pass absorption ratio is above ~90%). The absorbed power of ECH was estimated by the TRAVIS code^18^. The plasma current ramps up to 1.5 kA and is mainly driven by a bootstrap current. An electron cyclotron (EC) current was not driven because of the parallel refractive index of ${N}_{\parallel }=0.0$ of the injected EC waves.

### Nd:YAG Thomson scattering (YAG-TS) measurement

Thomson scattering of laser light is a popular method of measuring electron temperature (*T*_*e*_) and density (*n*_*e*_) profile in plasma. This method measures the scattering light due to electrons in the plasma. The *T*_*e*_ and *n*_*e*_ are obtained from the Doppler broadening and absolute intensity of scattering light, respectively. In this study, we measured the radial profiles for the  *T*_*e*_ and  *n*_*e*_ by means of the Nd:YAG Thomson scattering (YAG-TS) system^15–17^. The system consists of two 50 Hz lasers each of 550 mJ, large collection optics, and 25 radial channel interference polychromators (~10 mm spatial resolution). This measurement system achieves a S/N ratio of ~50 for low-density plasma (*n*_*e*_ ~ 0.5 × 10^19^ m^−3^).

### Electron cyclotron emission (ECE) radiometer

The intensity of electron cyclotron emission (ECE) from the plasma is proportional to *T*_*e*_ and ECE is widely used for a diagnostic of plasma electron temperature. The frequency of ECE corresponds to magnetic field strength in one-to-one, realising local measurement every frequency. The ECE system in Heliotron J measures *T*_*e*_ by using second harmonic X-mode at 16 radial points in one discharge. The eITB plasma which is target of this research (*n*_*e*_ ~ 1 × 10^19^ m^−3^, *T*_*e*_ ~ 2 keV) is optically thick to measure the temperature of bulk electron.
